# 

**Published:** 1911-03-25

**Authors:** 


### Publications.

**NOW READY.** New and greatly enlarged Edition, containing 415 Illustrations.

### TEXT-BOOK OF OPERATIVE SURGERY

BY

**Dr. THEODOR KOCHER**

AUTHORISED TRANSLATION FROM THE FIFTH GERMAN EDITION

BY

HAROLD J. STILES, M.B., F.R.C.S. Edin.

AND

C. BALFOUR PAUL, M.B., F.R.C.S. Edin.

**Price 30/- Net**

Published by

A. & C. BLACK, 4 SOHO SQUARE, LONDON

### CLINICAL LECTURES

By HENRY CURTIS, B.S., F.R.C.S.,

Senior Assistant Surgeon to the Metropolitan Hospital, and Assistant Surgeon to the Seamen's Hospital Society; Joint Teacher of Operative Surgery, London School of Clinical Medicine.

**CASES OF ABDOMINAL SURGERY,** including some Rare Forms of Intestinal Obstruction. Illustrations. Price **2s.** net.

**LIVER ABSCESSES:** Their Etiology, Symptoms, and Treatment. Price **1s.** net.

**ON GUMMATA:** The Clinical Features and Treatment of the less obvious varieties, with a Record of Eight Cases. Illustrated. (*In the Press.*)

**INTERESTING CASES OF CRANIAL SURGERY.** Illustrated. Price **2s.** net.

London: THE MEDICAL PUBLISHING CO., Ltd., 22½ Bartholomew Close, E.C.

ALSO BY THE SAME WRITER.

**MASTOID ABSCESSES AND THEIR TREATMENT.** Translated and Edited from the French of Profs. A BROCA, M.D., and LUBET-BARBON, M.D., Paris. With Coloured Illustrations, cloth, crown 8vo. **6s.**

London: H. K. LEWIS, 136 Gower Street, W.C.

Just Issued. Ninth Edition, Rewritten and Enlarged, **9s.** net.

### PHARMACY, MATERIA MEDICA & THERAPEUTICS.

Being a Commentary on the B.P. and its Drugs, with their Preparations, and on all the new Non-official Remedies in use, with Chapters on the different processes used in Dispensing, Prescription-writing, Latin Glossary, &c. By Sir W. WHITLA, M.D., LL.D., Professor, Materia Medica, Queen's University, Belfast, and Senior Physician, Royal Victoria Hospital.

By the same Author. New Work. 1,900 pages. Crown 8vo. **25s.** net.

### PRACTICE OF MEDICINE.

Containing in two Handy Volumes a complete and independent Article on the Etiology, Symptoms, and Diagnosis of every Medical Disease; arranged in Dictionary Form for Practitioners and Students.

London: BAILLIÈRE, TINDALL & COX, 8 Henrietta Street, W.C.

FIFTH EDITION. With 50 Illustrations. 5/- net.

### THE SCHOTT METHODS OF THE TREATMENT OF CHRONIC DISEASES OF THE HEART.

With an Account of the Nauheim Baths and of the Therapeutic Exercises.

By W. BEZLY THORNE, M.D., M.R.C.P.

London: J. & A. CHURCHILL, 7 Great Marlborough [illegible]

Life-Size Human

### ANATOMICAL MODEL

### (FEMALE).

**Being a reproduction of the Dissections of the various parts of the Human Body, showing the relations of the Internal Organs to one another. With a Descriptive Index.**

**By FREDK. NORMAN, F.R.C.S. Eng., and W. G. STONE, M.D. Oxon., F.R.C.S. Eng**

TO the Medical Man, the Student, the Nurse, or the Midwife, the value of this Model will be at once apparent. It will enable the various regions of the body to be viewed exactly as they would be seen in a succession of ideal dissections. The surface of the skin, the subcutaneous tissues with their blood vessels and nerves, the deeper tissues, the muscles or internal organs, and lastly the ligaments, joints and bones.

### THE MODEL'S MANY ADVANTAGES.

This Model is far and away better for practical purposes than book illustrations, which must necessarily be greatly reduced. A great inconvenience always experienced with book illustrations is the fact that one cannot turn rapidly from the study of one dissected area to that revealed in the next stage of dissection. By the use of this Anatomical Model the various regions can be viewed exactly as they would be seen in a series of dissections. Whilst in some ways inferior to actual dissections for special purposes it is more useful. For instance, it enables the student first to observe the anterior surface of an organ, and the parts in contact with it, and then to turn the organ over and view the under surface and the parts exposed by its removal.

**SUPPLIED FOR PROFESSIONAL OR SCIENTIFIC PURPOSES ONLY. . .**

THE HOSPITAL.—"This is an extremely useful and accurate model of dissections and should prove very suitable for demonstration work in classes . . . . We can thoroughly recommend it to those who are desirous of possessing a really sound and useful model."

FOLIA THERAPEUTICA says:—"A stereoscopic atlas is necessarily a scattered and diffuse collection of photographs. and it therefore fails to present a rapid, comprehensive, and bird's-eye view of the salient anatomical points required by the practitioner. This desideratum has, however, recently been supplied by The Gresham Publishing Company, who have prepared a life-size reproduction of the dissections of the various parts of the human body, in the form of a model, remarkably convenient in arrangement and admirably simple in manipulation."

THE LANCET.—"The model will prove of assistance to senior students and practitioners who may have occasion to review their anatomy, especially in regard to anatomical relations"

### ENQUIRY FORM.

To the GRESHAM PUBLISHING CO,
34-5 Southampton Street, Strand, London, W.C.

Please send me prospectus describing the LIFE-SIZE ANATOMICAL MODEL, and particulars of the terms on which you will supply it to readers of "THE HOSPITAL."

*Name*..........................................

*Address*..........................................

(Send this form in ½d. envelope, or pasted to a p.c.)

**Publications**—*continued.*

**By E. HURRY FENWICK, F.R.C.S.,** Professor of Urology at the London Hospital; President, Congress International Society of Urology.

**NEW WORK.** With 80 Plates and 15 Text Figures. **10s. 6d.** net.

### X RAY DIAGNOSIS OF URINARY STONE:

### AN OPERATIVE AND CLINICAL RECORD BASED ON 1,000 RADIOGRAPHS.

London: J. & A. CHURCHILL, 7 Great Marlborough Street, W.

---

**By A. H. TUBBY, M.S.**

### DEFORMITIES.

Six hundred pages, 300 Illustrations. Price **17s.**
"Standard work on the subject in the English language."
*British Medical Journal.*

### SURGERY OF PARALYSES.

**By A. H. TUBBY, M.S.Lond., F.R.C.S.Eng., and ROBERT JONES, F.R.C.S.E.**
Illustrated by 93 figures and 68 cases. Pp. 301. **10s. net.**
"Can confidently recommend it as a practical summary of the most modern views."—*Edinburgh Medical Journal.*
"Indicates original and successful methods of treatment."
*American Journal of Orthopœdic Surgery.*

London: Macmillan & Co., Ltd. New York: The Macmillan Co.

---

**THE IMMEDIATE TREATMENT OF INJURIES BY FRICTION AND MOVEMENT.**

By WHARTON P. HOOD, M.D. St. Andrews, M.R.C.S., L.S.A., &c.

Crown 8vo. 4s. 6d.

BRITISH MEDICAL JOURNAL.—"A careful reading of the book, which is very well written, will certainly be helpful to many surgeons."

LANCET.—"We can cordially recommend Dr. Hood's work to the attention of every practitioner who is liable to be called upon to deal with injuries. . . We believe that very few readers will either be inclined to lay it aside until they have finished it or will fail to obtain valuable practical suggestions from its pages."

MACMILLAN & CO., LTD., London.

---

W. CARNEGIE BROWN, M.D., M.R.C.P.

Just published.

### AMOEBIC or TROPICAL DYSENTERY

**Its Complications and Treatment.**
Fcap. 4to. with numerous Illustrations, price 7s. 6d. net.
Also

### SPRUE AND ITS TREATMENT.

Fcap. 4to. with 2 Plates, cloth, gilt lettered, price 6s. net.
"A book which should prove useful to the medical profession generally."
"Supplies important information. Valuable to tropical practitioners."
*Brit. Med. Jour.*
"The standard work on the subject."—*Medical Press.*
London: J. BALE, SONS & DANIELSSON, Ltd., Great Titchfield Street.

---

### WORKS BY DAVID WALSH, M.D.

### THE HAIR AND ITS DISEASES.

*Second Edition.* 2s. 6d. net. A Standard Monograph.
Baillière, Tindall & Cox, London.

### GOLDEN RULES OF SKIN PRACTICE.

1s. net. *Third Edition.* Wright & Co., Bristol.

---

### "The Prescriber."

**A Monthly Journal dealing with Therapeutics, Newer Remedies, &c.**

"One of the most useful journals the practitioner can subscribe to."
See *The Hospital*, Jan. 7, 1911.

**Five Shillings per annum**

**Write for a free Specimen Copy.**

**THE PRESCRIBER" Office, 137A George St., EDINBURGH.**

---

### EXAMINATIONS IN HYGIENE

These are held twice a year—in January and July—for the Certificate and Diploma of the Institute, as well as the following Honours Subjects, viz:—

| | |
|---|---|
| **The Hygiene of Motherhood.** | **Cooking.** |
| **The Feeding and Rearing of Children.** | **Food and Dietetics.** |
| | **The Hygiene of the Home.** |
| **Home Nursing and First Aid.** | **Physical Training.** |

The Examinations take place at all the chief towns throughout the United Kingdom. For Syllabus and further particulars address, *The Secretary*, **INSTITUTE OF HYGIENE,** 34 Devonshire St., Harley St, London, W.

---

SECOND EDITION JUST PUBLISHED.
Pp. xii + 210, with 120 Illustrations. Price **6s.** net.

### Handbook of Intestinal Surgery.

**By L. A. BIDWELL, F.R.C.S.Eng.,**
Surgeon, West London Hospital; Lecturer on Intestinal Surgery and Dean of the Post-Graduate College, &c.
London:
BAILLIÈRE, TINDALL & COX, 8 Henrietta Street, Covent Garden.

---

### INJURIES OF THE EYES OF THE EMPLOYED.

By W. M. BEAUMONT. 5s.
"An instructive guide."—*Brit. Med. Jour.* ". . . This handy, clever book." "The teaching is sound and trustworthy."—*Lancet.* *Medical Press.*
London: H. K. LEWIS.

---

**Price 3s. 6d.**

### CANCER.

**By G. SHERMAN BIGG, F.R.C.S.E.**
London: BAILLIÈRE, TINDALL & COX, 8 Henrietta St., Covent Garden.

---

***Price 2s. 6d.***

### CONSTIPATION.

**By G. SHERMAN BIGG, F.R.C.S.E.**
London: BAILLIÈRE, TINDALL & COX, 8 Henrietta Street, Covent Garden.

---

By HERBERT J. PATERSON, M.B., F.R.C.S.

### JEJUNAL AND GASTRO-JEJUNAL ULCER.

Just published. 2s. 6d. net.
JOHN BALE, SONS & DANIELSSON.

### GASTRIC SURGERY.

Price 6s. net.

---

### BODILY DEFORMITIES. Vol. I.

A Series of Lectures on their Nature, Causes, Variety and Treatment, delivered at the City Orthopædic Hospital by the late E. J. CHANCE, F.R.C.S.Eng. Edited by JOHN POLAND, F.R.C.S.Eng., Consulting Surgeon to the Miller General Hospital for South-East London, late Senior Surgeon to the City Orthopædic Hospital, &c. SECOND EDITION. With numerous Illustrations. 6s. net.

*THE POLYCLINIC.*—"Altogether Mr. Poland has given us a delightful volume, and the profession owes much to him for this opportunity of studying his late colleague's exact and thoughtful work."

☞ *Volume II., completing the work, is in the press.*
LONDON: SMITH, ELDER & CO., 15 WATERLOO PLACE, S.W.

---

**2s. 6d. net,** 8vo. cloth, pp. 104.

### POINTS of PRACTICE in MALADIES of the HEART.

The Three Lumleian Lectures at the Royal College of Physicians, 1908.
By **SIR JAMES SAWYER,** M.D. Lond., F.R.C.P., F.S.A.,
Senior Consulting Physician to The Queen's Hospital.
"Will amply repay reading by all medical men."—W. LONDON MED. JOUR.
BY THE SAME AUTHOR.
Fourth Enlarged Edition. **3s. net,** 8vo. cloth, pp. 227.

### CONTRIBUTIONS TO PRACTICAL MEDICINE.

Contents—Insomnia; Gastralgia; Lungs and Heart; Floating Kidney; Habitual Constipation; Intestinal Obstruction; Ung. Ranunc. Fic. in Piles; Eczema; Chorea; Phthisis; Medicated Lozenges; Inhalations in Asthma; Diabetic Diet, &c.
"Conveying much practical information."—THE LANCET.

Separately. **1s. 3d. net,** 8vo. cloth, pp. 66.

### INSOMNIA: ITS CAUSES AND CURE.

Two Clinical Lectures.
"Indications for treatment excellent."—ST. THOS. HOSP. GAZ.

Birmingham: CORNISH BROTHERS, Limited.

Publications—*continued.*

### CASSELL'S MEDICAL WORKS.

**JUST PUBLISHED.**

*New Edition, Revised Throughout.* *686 pages, demy 8vo. 21s. net.*

### TUMOURS, INNOCENT AND MALIGNANT:

**THEIR CLINICAL CHARACTERS AND APPROPRIATE TREATMENT.**

By J. BLAND-SUTTON, F.R.C.S.,

Surgeon to, and Member of the Cancer Investigation Committee of, the Middlesex Hospital, &c.

This work has been thoroughly revised by the Author, much new matter has been incorporated, and many new Illustrations have been added. The Author carefully discusses the Etiology of Cancer in the light of the results of the most recent investigations. *Prospectus post free.*

AN ENTIRELY NEW WORK.
A TEXT-BOOK OF

### Gynæcological Surgery

— BY —

COMYNS BERKELEY, M.A., M.D., B.C. Cantab., F.R.C.P. Lond., M.R.C.S. Eng.,

AND

VICTOR BONNEY, M.S., M.D., B.Sc. Lond., F.R.C.S. Eng., M.R.C.P. Lond.

With 392 Figures in the Text from Drawings by VICTOR BONNEY, and 16 Coloured Plates. 720 pp., demy 8vo, 25s. net. *Prospectus post free.*

### The Diagnosis of Smallpox

— BY —

T. F. RICKETTS, M.D., B.Sc. Lond., M.R.C.P., D.P.H.,

Medical Superintendent of the Smallpox Hospitals and of the River Ambulance Service of the Metropolitan Asylums Board.

Illustrated from Photographs by J. B. BYLES, M.D., B.C. Cantab., F.R.C.S. Eng., D.P.H. 12 Coloured and 110 Black-and-White Plates, and 14 Charts. 21s.

"The work is one of those, unless we are much mistaken, which are destined to occupy a leading place for many years to come in the literature of epidemiology."—*Lancet.*

CASSELL & CO., LTD., La Belle Sauvage, London, E.C.

### WORKS BY SIR LAUDER BRUNTON, Bt., M.D., &c.

### THERAPEUTICS OF THE CIRCULATION.

Eight Lectures delivered in the Physiological Laboratory of the University of London. Published under the auspices of the University of London. 7s. 6d. net.

JOHN MURRAY, LONDON.

### COLLECTED PAPERS ON CIRCULATION AND RESPIRATION.

First Series, chiefly experimental, 8vo. 712 pp. 7s. 6d. net.

**A TEXT-BOOK OF PHARMACOLOGY, THERAPEUTICS, AND MATERIA MEDICA.** Adapted to the United States Pharmacopœia by F. H. WILLIAMS, M.D., Boston, Mass. Third Edition, containing the Additions, 1891, to the British Pharmacopœia. 8vo. 21s. In 2 Vols. 22s. 6d.

**ON DISORDERS OF DIGESTION:** their Consequences and Treatment. 8vo. 10s. 6d.

**ON DISORDERS OF ASSIMILATION, DIGESTION, &c.** Illustrated. 8vo. 10s. 6d. net.

**LECTURES ON THE ACTION OF MEDICINES:** Being the Course of Lectures on Pharmacology and Therapeutics delivered at St. Bartholomew's Hospital during the Summer Session of 1896. 8vo. 10s. 6d. net.

MACMILLAN & CO., LTD., LONDON.

Fourth Edition. Entirely Revised and largely Re-written. 3 Coloured Plates and 215 Illustrations. 14s. net.

### DISEASES OF THE EAR,

Including the Nose and Throat in Relation to the Ear. By THOMAS BARR, M.D., and J. STODDART BARR, M.B., Lecturer and Assistant Lecturer on Diseases of the Ear, Glasgow University.

THE LANCET.—"It is a splendid text-book and admirably written."

THE JOURNAL OF LARYNGOLOGY, RHINOLOGY AND OTOLOGY.—"The various coloured illustrations are models of accuracy and beauty."

Glasgow: J. MACLEHOSE & SONS. London: MACMILLAN & CO.

### HEART DISEASE IN CHILDHOOD AND YOUTH.

By CHARLES W. CHAPMAN, M.D., M.R.C.P., Senior Physician to the National Hospital for Diseases of the Heart, Soho Square.

With an Introduction by SIR SAMUEL WILKS, Bart., M.D., F.R.S., Physician Extraordinary to H.M. the late Queen, ex-Pres. Roy. Coll. of Physicians.

Second Edition, with Two Appendices. Price 3s. 6d. post free.

London: THE MEDICAL PUBLISHING CO., 22½ Bartholomew Close, E.C.

### A GUIDE TO SICK NURSING IN THE TROPICS.

By ANDREW DUNCAN, M.D., B.S.Lond., F.R.C.P., F.R.C.S., Lt.-Col., I.M.S. (ret.), Joint Lecturer on Tropical Diseases at London School of Tropical Medicine; Lecturer on Tropical Diseases, Westminster Hospital; Fellow of King's College.

"The book should be most useful as a guide to those Nurses who intend offering for foreign service."—NURSING MIRROR.

Price 2s. 6d. THE SCIENTIFIC PRESS, LTD.

BY THE SAME AUTHOR.

### THE PREVENTION OF DISEASE IN TROPICA CAMPAIGNS.

Founded on the Parkes' Memorial Prize Essay for 1886.

"We have no hesitation in saying that Dr. Duncan's book is of great value to the State, indispensable to the Army Surgeon, and highly creditable to the author."—BRIT. MED. JOURNAL.

"In the book before us we find in a compact form, and easy of reference, most of the information required in an ordinary tropical campaign."
EDIN. MED. JOURNAL.

"If we mistake not, this will form the favourite text-book with young Army Surgeons."—ILLUSTRATED MEDICAL NEWS.

Price 12s. 6d. J. & A. CHURCHILL.

### Publications—*continued.*

By W. McADAM ECCLES, M.S., F.R.C.S.

**HERNIA.** Third Edition, 1908.

Price 7s. 6d. net. 121 Plates and other Illustrations.

**THE IMPERFECTLY DESCENDED TESTIS.**

Embodying the JACKSONIAN PRIZE ESSAY. Price 7s. 6d. net.
41 Plates and 76 Figures.

London :
BAILLIÈRE, TINDALL & COX, 8 Henrietta Street, Covent Garden.

---

Pp. xii+583. With 77 Illustrations. Price 10s. 6d. net.

**DICTIONARY OF MEDICAL DIAGNOSIS.**

**A Treatise on Signs and Symptoms observed in Diseased Conditions.**

By HENRY LAWRENCE McKISACK, M.D., M.R.C.P.Lond.,
Physician to the Royal Victoria Hospital, Belfast.

"It is well written and refreshingly original."—*Dublin Medical Journal.*

London : BAILLIÈRE, TINDALL & COX, 8 Henrietta Street, Covent Garden.
Dublin : HANNA & NEALE, 18 Nassau Street.

---

"The best book on the subject we have seen."—*The Journal of the Royal Army Medical Corps.* Demy 8vo. 554 pages. **9s.** net.

**A SYSTEM OF SURGICAL NURSING** (**With an Appendix containing useful Formulæ, Emergency Drill, &c.**)

By A. N. M'GREGOR, M.D., F.F.P.S.G., *Assistant Surgeon to the Glasgow Royal Infirmary.*

**Extracts from some Press Opinions.**

"Is one of the best of its kind. The teaching is good, and many useful hints are given on points which should be known to all skilfully trained nurses."—**The Lancet.**

"There seems to be no detail omitted, however small, that concerns the comfort and well-being of the patient."—**The Nursing Times.**

"The outcome of much labour, and should stand as a work of reference in the house-surgeon's room, in the matron's office, and in the library of every nursing home."

**The Scottish Medical and Surgical Journal.**

"We can cordially recommend Dr. M'Gregor's book as one which can be offered to nurses as a standard of good teaching, containing thoroughly up-to-date principles as practised in large hospitals. . . ."

**The Caledonian Medical Journal.**

"No subject appears to have been neglected or overlooked. . . . We can recommend this book as a complete and reliable Manual of Nursing.'

**The Nurses' Journal.**

"The author has undertaken a big task, and has successfully performed it."—**Nursing Notes.**

**GLASGOW: DAVID BRYCE & SON.**

---

9th Edition. Text 1,044 pp., with 127 Coloured and Plain Plates, and 653 other Illustrations. In one vol. Price in two vols. 22s. 6d. net.

**MANUAL OF DISEASES OF WOMEN.**

By H. MACNAUGHTON-JONES,
M.D.Q.U.I., M.Ch., M.A.O., F.R.C.S I. & E.
President of the Obstetrical and Gynæcological Section of the Royal Society of Medicine; formerly University Professor and Examiner in Midwifery and Diseases of Women and Children in the Queen's and Royal Universities of Ireland.

London : Baillière, Tindall & Cox, 8 Henrietta St., Covent Garden.
Dublin : Hanna & Neale, 18 Nassau Street, and all Booksellers.

---

A MANUAL OF

**MEDICAL EXERCISES.**

By PERCY LEWIS, M.D., M.R.C.S.,
Hon. Medical Officer to Victoria Hospital, Folkestone.

66 pages, with 35 Illustrations, cloth, **1s. 6d.** net; post free, 1s. 7d.

H. K. LEWIS, 136 GOWER STREET, LONDON, W.C.

---

Just Published. Crown 8vo. Price 1s. net.

By post 1s. 1d.

THE

**Non-Surgical Treatment of Duodenal Ulcer.**

**By George Herschell, M.D., Lond.**

LONDON :
H. J. GLAISHER, 55-57 Wigmore Street,
Cavendish Street, W.

With Coloured Diagrams, demy 8vo. **6s.**

**ON THE PRINCIPLES WHICH GOVERN TREATMENT IN DISEASES AND DISORDERS OF THE HEART.**

(Being the Lumleian Lectures, 1899.)

By Sir RICHARD DOUGLAS POWELL, Bart., K.C.V.O., M.D.Lond., F.R.C.P.

London: H. K. LEWIS, 136 Gower Street, W.C.

---

**A PRACTICAL GUIDE**
TO
**SURGICAL BANDAGING AND DRESSINGS.**

A Pocket Book useful to the Student.
**2s.** post free.

THE SCIENTIFIC PRESS, Ltd., 28 & 29 Southampton St., Strand, W.C

### BOOKS RECEIVED.

Local Government Journal.
"Local Government Annual," 1911.

J. Wright and Sons.

"Golden Rules of Ophthalmic Practice." By G. Hartridge.

J. and A. Churchill.
"Urine Examination." By Carruthers.
"Vicious Circles in Disease." By. J. B. Hurry.

Critic and Guide Co.
"Prevention of Sexual Diseases." By Victor C. Vecki, M.D.

James Thin.
"A Thrifty Hospital." By W. E. Home, R.N.

Henry Frowde, and Hodder and Stoughton.
"Cholera and its Treatment." By L. Rogers.

Cassell and Co.
"Gynæcological Surgery." By C. Berkeley and V. Bonney.

W. Green and Sons.
"The Cancer Problem." By C. E. Green.

Putnam and Sons.
"The Story of the Bacteria." By Prudden (T. M.).

At the meeting of the South Paddington District Branch of the League of Mercy, held last week at Lancaster Gate, Sir Dyce Duckworth defended the voluntary system. His claims on its behalf were illustrated by the figures brought forward by Prebendary Francis Gurdon, who presided, and reminded the meeting that the League handed over £18,000 last year to King Edward's Hospital Fund.

### THE INSTITUTE OF HUMAN PALÆONTOLOGY.

The Institute of Human Palæontology, a new foundation due to the liberality of the Prince of Monaco, and recently recognised as being of public utility, will have for its aim the study of the origins of humanity and the methods of research for the purpose of clearing up and establishing the still doubtful history of the more ancient civilisations. The administrative council will include six members of French nationality, with whom will work a committee of improvement composed of French and foreign scientists. A building is to be erected in Paris to serve for social gatherings, but the collection of specimens will be housed at Monaco. The Institute is in command of capital amounting to £64,000.

### Obituary.

Dr. John Attfield, who died at Walford on March 18, was best known to medical practitioners as Editor of the British Pharmacopœia and the Indian and Colonial Addendum, and to medical students as the author of "Chemistry: General, Medical, and Pharmaceutical," of which the first edition appeared in 1867. He was born in 1835, and became a student in the Pharmaceutical Society's School in 1853; nine years later he was appointed professor of practical chemistry in that school, a position which he held until 1896. He was one of the founders of the British Pharmaceutical Conference and of the Institute of Chemistry, and was a Fellow of the Royal Society.

### COMING EVENTS OF THE WEEK.

[Communications for this column must be sent to 29 Southampton Street, Strand, before noon on Wednesday.]

Monday, March 27: League of Mercy (City District): Annual meeting, the Lord Mayor presiding, Mansion House, 4 p.m. Speakers, the Right Hon. The Lord Farquhar, Sir Frederick Treves, Bart., G.C.V.O., C.B., etc., Sir William Collins, M.D., F.R.C.S.

Tuesday, March 28: Sick Room Helps' Society and Nurses' Home: Annual meeting, Sir Herbert Cohen presiding, 3 Hamilton Place, 3.15 p.m.
Annual General Meeting of St. Peter's Hospital subscribers, at 5 o'clock p.m., when Mr. F. A. Bevan, the Treasurer, will preside.

Wednesday, March 29: Mr. and Mrs. Fewlass Llewellyn and their friends are giving an entertainment to the patients of the Royal Hospital for Incurables, Putney.

Thursday, March 30: Annual meeting of Governors of Chelsea Hospital for Women, 4 p.m. Viscount Castlereagh, M.P., M.V.O., President, in the chair.

Friday, March 31: The annual general meeting of the Medical Graduates' College and Polyclinic will be held at 22 Chenies Street, London, W.C., at 5.15 p.m.

### UNIVERSITY EDUCATION IN LONDON.

A further Blue-book, consisting of minutes of evidence with appendices, has been issued in connection with the Royal Commission on university education in London. Part of the evidence of Dr. W. D. Halliburton, Professor of Physiology at King's College, relates to medical education. Asked whether it was possible, in the case of the great medical schools, to separate the hospital management from the teaching, making them schools of medicine and bringing them into the university, Professor Halliburton said that between the London hospitals there was a certain amount of rivalry, and the difficulty had always been to smooth over such difficulties and make the hospitals work together to a common end. Asked by the chairman, Mr. R. B. Haldane, whether he saw any intrinsic difficulties, for instance in the scientific teaching at Guy's being made part of the university, either as a school or incorporated, and the hospital kept closely connected with it, but not incorporated, Professor Halliburton said he thought it would be an extremely good thing, provided that the university did not undertake the hospital management. Mr. Haldane suggested that such an arrangement might possibly facilitate the assistance of these hospitals from the King's Hospital Fund, where there had been very serious question as to how far the fund may be properly applied in the promotion of teaching. In a short note in one of the appendices Mr. Leonard Hill says: "Frequent tests by examination, in my opinion, ruin the individuality of the teacher, prevent his influence on the character of the student, and rob the student of originality, self-reliance, and power of independent inquiry. In the medical curriculum, for example, there ought, in my opinion, to be only one test, a final searching examination into a man's fitness to be qualified to practise. No students should enter the wards from the start and receive their scientific training and their clinical experience side by side during the whole five and a half years of the curriculum."—"Royal Commission on University Education in London—Appendix to Second Report," Cd. 5528, price 3s. 8d.

### The Hospital World.

### Jottings.

On Tuesday, March 14, Sir George White, the President, laid the foundation stone of the new Surgical wards of the Bristol Royal Infirmary, which are to be erected near the original buildings. The new premises, which will include 180 additional beds and three operating theatres, will be a memorial to the late King and will be called "The King Edward Memorial Infirmary." £70,000 is needed, and £35,000 is in hand.

At the annual meeting of the Leicester Saturday Hospital Society it was stated that the names of sixty-three firms employing 1,916 workers were added to the contributors during the year 1910, and that the total income of the Society was £13,791. By arrangement 70 per cent. of this total sum (less expenses) went to the Leicester Infirmary and Children's Hospital. The new Home for Women at Woodhouse Eaves is making rapid progress, and the Society hopes to hold its next annual meeting there. The Committee acknowledges the services of Dr. Gordon Kelly, the Medical Officer, and Miss Havers, the Matron at Desford, as well as the unremitting services of Mr. H. H. Woolley, the Organising Secretary. Sir Edward Wood was re-elected President.

### Gifts and Bequests.

Hospital treasurers will be interested to learn that Mr. George Bell, North Claughton, Birkenhead, formerly a shipowner, has left the residue of his estate, which will probably amount to £10,000, to any charitable institutions which his trustees shall determine.

Mrs. Martha Beatrice Jefferies, of Grasmere, Westmorland, who died on January 26, who left estate valued at £30,255 gross, has bequeathed £100 each to the Royal Hospital for Incurables, Putney, and to the Kendal County Hospital.

The Royal Hospital for Incurables, Putney Heath, recently appealed through the newspapers for a gift of an organ for the large Assembly Room. Mr. and Mrs. Lesslie, of South Croydon, have generously made a gift to this National Charity of a suitable instrument.

Mr. John Vicary, of Ealing, W., left £500 each to the Devon and Exeter Hospital, the Orphan Asylum, Watford, and the Home for Incurables, Putney. He left the residue of his property, about £20,000, for charities in or connected with the City or County of London.

Mr. William Taylor, of Eastbourne, and formerly of Messrs. W. R. Taylor and Co., paint manufacturers, Liverpool, who left estate of the gross value of £94,968, has bequeathed, subject to the life interest of his wife: £2000 to the Liverpool Royal Infirmary; £2000 to the Royal Hospital for Incurables, Putney Heath, S.W.; £2000 to the Hospital for Consumption and Diseases of the Chest, Fulham Road, S.W.; £2000 to the Cancer Hospital, Fulham Road, S.W.; £2000 to the Royal Free Hospital, Gray's Inn Road, W.C.; £2000 to the Home for Confirmed Invalids, Aubert Park, Highbury, N.; and £1000 to the Samaritan Free Hospital for Women, Marylebone Road, W. He also left £21,000 upon trust for certain relatives in each case for life, and subject thereto. These sums are to be divided equally between abovementioned institutions.

Mr. Edwin James Oates, of South Royde, Halifax, worsted spinner, who left estate valued at £152,698 gross, has bequeathed £1000 to the Royal Halifax Infirmary.

### APPOINTMENTS VACANT.

Full particulars of these vacancies can be obtained on reference to our advertisement columns:—

Devonshire Hospital, Girton.—Pathologist.
Joint Counties Asylum, Carmarthen.—Second Assistant Medical Officer.
Parish of Birmingham.—Resident Medical Officer.
Seamen's Hospital Society.—Two House Physicians, Two House Surgeons.
Warneford Hospital, Leamington.—Junior Resident Medical Officer.
West Bromwich District Hospital.—Senior House Surgeon.
Western General Dispensary.—Honorary Surgeon.
West Ham and Eastern General Hospital.—House Surgeon, Junior House Physician.
Wolverhampton General Hospital.—House Surgeon.

